# The effect of compressive loading magnitude on in situ chondrocyte calcium signaling

**DOI:** 10.1007/s10237-014-0594-4

**Published:** 2014-05-23

**Authors:** Ryan M. J. Madden, Sang-Kuy Han, Walter Herzog

**Affiliations:** 1 Human Performance Laboratory, University of Calgary, 2500 University Dr. NW, Calgary, AB T2N 1N4 Canada; 2 Advanced Biomedical & Smart Welfare Technology R&BD Group, Korea Institute of Industrial Technology, Cheonan-si, 331-822 Chungcheongnam-do Republic of Korea

**Keywords:** Articular cartilage, Chondrocyte, Calcium signaling, Mechanobiology, Mechanotransduction

## Abstract

Chondrocyte metabolism is stimulated by deformation and is associated with structural changes in the cartilage extracellular matrix (ECM), suggesting that these cells are involved in maintaining tissue health and integrity. Calcium signaling is an initial step in chondrocyte mechanotransduction that has been linked to many cellular processes. Previous studies using isolated chondrocytes proposed loading magnitude as an important factor regulating this response. However, calcium signaling in the intact cartilage differs compared to isolated cells. The purpose of this study was to investigate the effect of loading magnitude on chondrocyte calcium signaling in intact cartilage. We hypothesized that the percentage of cells exhibiting at least one calcium signal increases with increasing load. Fully intact rabbit femoral condyle and patellar bone/cartilage samples were incubated in calcium-sensitive dyes and imaged continuously under compressive loads of 10–40 % strain. Calcium signaling was primarily associated with the dynamic loading phase and greatly increased beyond a threshold deformation of about 10 % nominal tissue strain. There was a trend toward more cells exhibiting calcium signaling as loading magnitude increased ($p$ = 0.133). These results provide novel information toward identifying mechanisms underlying calcium-dependent signaling pathways related to cartilage homeostasis and possibly the onset and progression of osteoarthritis.

## Introduction

Osteoarthritis (OA) is a degenerative joint disease characterized by a breakdown of the articular cartilage extracellular matrix (ECM) resulting in joint pain, inflammation, and stiffness. Currently, the mechanisms underlying the onset and progression of OA are not well understood. Under normal physiological conditions, joint loading causes deformation of the cartilage and its cells (Abusara et al. 2011). This deformation causes a complex array of biological processes to occur resulting in the synthesis and/or degradation of structural macromolecules (Sah et al. 1989). These processes have been associated with the adaptive/degenerative changes involved in OA (Wieland et al. 2005). Despite this knowledge, the complex processes behind cell mechanotransduction remain to be elucidated. One of the initial steps is an influx of calcium into the cell cytoplasm known as calcium signaling (Guilak et al. 1999). Calcium is a second messenger directly involved in many cellular processes including gene transcription, contraction, and proliferation (Bootman et al. 2001). It can enter the cytoplasm from extracellular stores through the activation of various membrane channels or from intracellular stores, such as the endoplasmic reticulum, which releases calcium via channels such as the inositol-1,4,5-triphosphate (IP3R) receptor (Berridge 1993; Berridge et al. 2003; Huser and Davies 2007).

For chondrocytes, calcium is involved in matrix synthesis (Clark et al. 1994; Valhmu and Raia 2002), cytoskeletal remodeling (Erickson et al. 2003; Millward-Sadler and Salter 2004), cell hyperpolarization (Wright et al. 1996), and cell death (Huser and Davies 2007; Amin et al. 2009). Previous work investigating chondrocyte calcium signaling has focused on cells removed from their physiological environment, such as isolated cells/cell cultures (Yellowley et al. 1997; Guilak et al. 1999; Mizuno 2005; Chao et al. 2006; Kono et al. 2006), and chondrocytes embedded in gel constructs (Roberts et al. 2001; Pingguan-Murphy et al. 2005, 2006). It was recently shown that calcium signaling of chondrocytes in situ differs compared to isolated cells embedded in gel constructs. Specifically, calcium signaling in situ occurred virtually instantaneously with loading as compared to the time-delayed signaling previously observed in cell-gel constructs (Han et al. 2012). The duration of calcium signals observed in in situ chondrocytes was also shorter than signals observed in cell-gel constructs and completely isolated cells and instead were closer to the durations of osmotically induced calcium signals in intact mouse femora (Han et al. 2012). These results suggest that the calcium signaling behavior of chondrocytes in the intact tissue differs substantially from isolated cells and cell-gel constructs. Therefore, further investigation is needed to understand chondrocyte mechanotransduction as it occurs in intact cartilage.

Loading magnitude is thought to play a key role in the calcium signaling response of chondrocytes (Yellowley et al. 1997; Guilak et al. 1999), and the magnitude of mechanical stimuli has been shown to greatly influence Ca^2+^ signaling in other cell types, such as endothelial cells (Shen et al. 1992) and osteocytes (Lu et al. 2012). We have shown previously that local ECM strains and chondrocyte deformations induced by mechanical loading differ between joint regions (Madden et al. 2013) and others have observed topographical variations in aggrecan synthesis (Little and Ghosh 1997), a process that involves calcium signaling (Fitzgerald et al. 2004). Therefore, the purpose of this study was to examine the effect of compressive loading magnitude on chondrocyte calcium signaling in intact cartilage attached to its native bone for two distinct joint regions. A secondary objective was to relate previously measured local ECM strains and chondrocyte deformations (Madden et al. 2013) to the observed calcium response. We hypothesized that the percentage of cells exhibiting at least one calcium response would increase with increasing compressive tissue load.

## Methods

### Sample preparation

Cartilage samples were obtained from the knees of six to eight-month-old New Zealand White rabbits ($n$ = 5). All experiments were approved by the Animal Ethics Committee of the University of Calgary. Femoral condyles ($n$ = 10) and patellae ($n$ = 5) were extracted from the knee joints, stripped of non-cartilaginous connective tissues while maintaining the underlying bone, and placed in high glucose Dulbecco’s Modified Eagle’s Medium with 4 mM L-glutamine and 25 mM HEPES (DMEM, Gibco, OR, USA) supplemented with 1 % penicillin/streptomycin and 1 mM sodium pyruvate. Tissue thickness of each sample was measured by needle indentation at two locations close to the region to be loaded. Cartilage samples were then incubated in medium containing the ratiometric calcium-sensitive dyes Fura Red (30 $\upmu$M) and Fluo-4 (15 $\upmu$M) for 1 h at 37 $^\circ$C. After staining, the samples were rinsed in dye-free medium for 20 min prior to testing. All specimens were tested within 15 h of collection, and the order of testing was randomized to mitigate the effect of time.

### Mechanical testing and confocal imaging

**Fig. 1:**
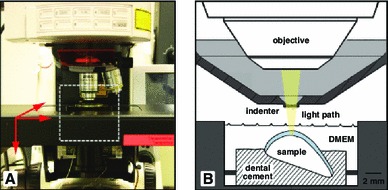
**a** Assembled view of the system for simultaneous loading and imaging of in situ chondrocytes. **b** Schematic of *dashed white box* shown in **a** showing the orientation of the sample in the tissue holder, the indenter, and the light path of the confocal microscope

**Fig. 2:**
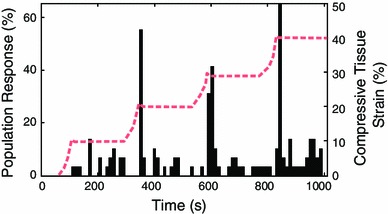
*Black bars* percentage of cells exhibiting a calcium signal for a representative sample (1 bar = 3 image frames = 12 s). The *dashed red line* shows the applied loading protocol on the secondary $y$ axis

Samples were embedded in dental cement and placed into the chamber of a custom-designed in situ indentation system (Fig. 1) mounted to the stage of a confocal laser scanning microscope (LSM 510, Zeiss Inc., Germany). Briefly, the system consists of a light-transmissible indenter, piezo-actuator, load cell, and displacement transducer (Han et al. 2009). The specimen height was adjusted to the point of initial contact between the indenter and cartilage surface, and samples were positioned as described previously (Han et al. 2010, 2012; Madden et al. 2013). A series of static loads were applied to the samples in the following order: 10, 20, 30 and 40 % compressive strain (Fig. 2). Loading was applied using a light-transmissible cylindrical indenter (diameter = 2 mm) at an average rate of 0.4 $\pm$ 0.2 %/s (1.8 $\pm$ 1.1 $\upmu$m/s) and controlled by a custom-written program (LabVIEW, National Instruments, USA). After the loading ramp was complete, displacements were held for three minutes as previous work has shown that the majority of in situ chondrocyte calcium signaling occurs during and immediately following the loading ramp (Han et al. 2012).

**Fig. 3:**
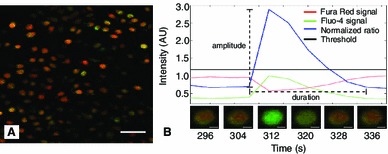
**a** Exemplar field of view (x–y plane) showing fluorescently labeled cells (*scale bar* = 50 $\upmu$m). **b** Calcium signal of a representative cell indicating the cell-specific threshold, signal characteristics, and corresponding confocal images (*scale bars* = 5 $\upmu$m)

Prior to loading, a characteristic group of cells was identified in the field of view and its position marked on the screen. This was done to ensure that the same cells were imaged throughout the entire loading protocol. Confocal images were captured through a 40x 0.8 NA 0.17 mm cover glass-corrected water immersion objective (Zeiss Inc., Germany). The image volume collected was 297 $\times$ 297 $\times$ 10 $\upmu {\text {m}}^3$ at a resolution of 0.58 $\upmu$m/pixel in the x–y plane (Fig. 3a). Confocal time series images were collected continuously at 0.25 Hz starting 60 s before application of the first load (eg. $t$ = 0 s in Fig. 2) until completion of the holding period of the final load (eg. $t$ = 1000 s in Fig. 2). The number of superficial zone chondrocytes imaged was 82 $\pm$ 6 (min: 37, max: 108) and 92 $\pm$ 7 (min: 69, max: 106) for femoral condyles and patellae samples, respectively. Specimen temperature was maintained at 37 $^\circ$C for the duration of the experiments.

### Confocal image analysis

The ratio of fluorescence intensity between Fura Red and Fluo-4 was normalized to the central moving average for each chondrocyte using a 60 s window. Calcium signals were defined as an increase in baseline levels greater than 10 times the standard deviation of the normalized ratio (Fig. 3b) (Han et al. 2012). The duration and peak amplitude of each calcium signal was determined. Response time was defined as the time from the onset of the loading ramp to the frame in which the most calcium signaling peaks were observed. This was done at each load and for each sample to compare the time response of calcium signaling as a function of loading magnitude. The population response of a sample was defined as the percentage of cells exhibiting at least one calcium signal over a specified period. The fraction of signals occurring at each load was expressed as a percentage of the total number of signals that occurred over the duration the experiment.

### Statistical analysis

Population response, signal fraction, and time response ($n$ = 10 femoral condyles and $n$ = 5 patellae) were examined using two-way repeated-measures ANOVA (SPSS 20, SPSS Inc., IL, USA) with joint region and load as the between- and within-subject factors, respectively ($\alpha$ = 0.05). Calcium signal amplitude and duration ($n$ = 946 signals from the 10 femoral condyles and $n$ = 751 signals from the 5 patellae) were analyzed using generalized estimating equations to determine the effect of joint region, loading magnitude, and interaction effects ($\alpha$ = 0.05) while accounting for multiple responses from the same cells and to account for the unbalanced design. Bonferroni post-hoc adjustments were conducted when appropriate. Results are presented as means $\pm$1 standard error of the mean.

## Results

### Population response

**Fig. 4:**
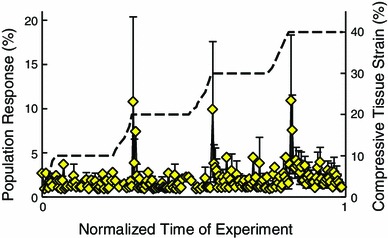
Average calcium signaling response of all femoral condyle samples over the duration of the experiments. The *dashed black line* shows the applied load on the secondary $y$ axis. Results are presented as means $\pm$1 standard error of the mean

**Fig. 5:**
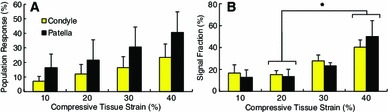
**a** Average calcium signaling response of all femoral condyle and patellar samples during each loading phase. **b** Fraction of signals occurring at each load level relative to the total number of signals occurring for each joint region (946 signals from 10 femoral condyles, 751 signals from 5 patellae). Results are presented as means $\pm$1 standard error of the mean. *Star* indicates a significant difference in pooled samples ($p<$ 0.05)

Figure 4 shows the population response of in situ chondrocytes over the entire experiment. During the initial 60 s unloaded imaging period, little to no calcium signaling activity was observed for all samples. Compressive loading of cartilage resulted in a calcium signal response in 32 $\pm$ 9 % of femoral condyle cells and 58 $\pm$ 16 % of patellar cells. The majority of calcium signals occurred during or immediately following the transient phase of loading (Fig. 4). The average response time to peak calcium signaling was 77 $\pm$ 8 s and 74 $\pm$ 10 s for femoral condyles and patellae, respectively, and generally decreased with increasing compressive tissue strain (Table 1). Figure 5a shows the population response of the chondrocytes at each load, defined as any cell that signaled between the start of the loading ramp for the load of interest and before the start of the next loading ramp. The percentage of chondrocytes responding with at least one calcium signaling event tended to increase with increasing load for both joint regions (Fig. 5a; Table 1). Patellar samples exhibited a trend toward greater population response at each load compared to the femoral condyles, although these differences did not reach statistical significance ($p>$0.05; Fig. 5a). For a given load, more responding chondrocytes exhibited a single calcium signaling event compared to multiple signals, except for the patellar samples at 20 % tissue strain.

While the main effect of loading magnitude did not reach statistical significance, there was a trend toward an increase in the population response with increasing load ($p$ = 0.133, Fig. 5a). Additionally, more calcium signaling events occurred at 40 % tissue strain; this difference was significant when compared to the 20 % tissue strain condition ($p<$ 0.05, Fig. 5b) and was close to significance compared to the 10 and 30 % tissue strain conditions ($p$ = 0.142 and 0.120, respectively).

### Calcium signal characteristics

**Fig. 6:**
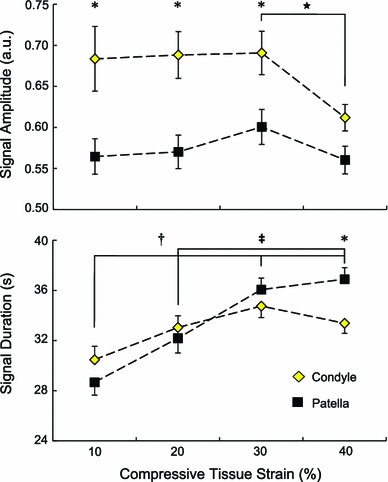
Calcium signal characteristics for chondrocytes from femoral condyles (*yellow diamonds*, $n$ = 946 signals from 10 samples) and patellae (*black squares*, $n$ = 751 signals from 5 samples). Results are presented as means $\pm$1 standard error of the mean. *Asterisk* indicates a significant difference between femoral condyles and patellae at a given load ($p<$ 0.05). *Star* indicates a significant difference within femoral condyles between the indicated loads ($p<$ 0.05). $\dagger$ and $\ddagger$ indicate significant differences within patellae between the indicated loads ($p<$ 0.001 and 0.05, respectively)

Both loading magnitude and joint region had a significant effect on calcium signal amplitude ($p<$ 0.05 and $p<$ 0.001, respectively). The calcium signal amplitude of femoral condyle chondrocytes remained consistent for the 10–30 % tissue strain conditions, but then decreased significantly at 40 % strain ($p<$ 0.05, Fig. 6). No significant change in signal amplitude was observed for patellar chondrocytes across all tissue strain conditions (10–40 %), although there was a slight decrease at 40 % strain, similar to the finding made for the femoral condyle cells. The signal amplitude of patellar cells was significantly less than femoral condyle cells for the 10–30 % tissue strain conditions ($p<$ 0.05, Fig. 6) and close to significance at 40 % ($p$ = 0.052).

Loading magnitude had a significant effect on the duration of calcium signals ($p<$ 0.001). For femoral condyle cells, signal duration remained relatively constant across all tissue loads, with a near-significant increase in signal duration at 30 % compared to 10 % tissue strain ($p$ = 0.050). In contrast, patellar chondrocytes exhibited steadily increasing signal durations as the tissue strain was increased (Fig. 6; Table 1). At 40 % tissue strain, the average calcium signal duration of patellar cells was significantly greater than that measured for femoral condyle cells ($p<$ 0.05, Fig. 6).

**Table 1:** Summary of calcium signaling event data obtained from mechanical compression tests of *in situ* chondrocytes

	Joint	Applied tissue strain (%, compressive)			
	Region	10	20	30	40
Population response$^\mathrm{a}$	COND	7.1 $\pm$ 3.3	12.1 $\pm$ 6.5	16.4 $\pm$ 7.6	23.5 $\pm$ 9.2
(%)	PAT	16.4 $\pm$ 9.4	21.6 $\pm$ 13.9	30.6 $\pm$ 13.7	40.6 $\pm$ 14.2
Signal fraction$^\mathrm{b}$	COND	16.7 $\pm$ 7.6	15.3 $\pm$ 3.6	27.8 $\pm$ 5.4	40.3 $\pm$ 6.6
(%)	PAT	12.9 $\pm$ 6.7	13.6 $\pm$ 6.5	23.3 $\pm$ 2.9	50.2 $\pm$ 14.3
Response time to peak$^\mathrm{a}$	COND	93 $\pm$ 22	73 $\pm$ 20	78 $\pm$ 12	69 $\pm$ 4
(s)	PAT	66 $\pm$ 15	80 $\pm$ 26	69 $\pm$ 19	80 $\pm$ 21
Local ECM strain$^\mathrm{c}$	COND	15 $\pm$ 2	23 $\pm$ 2	28 $\pm$ 2	34 $\pm$ 2
(% compressive)*	PAT	16 $\pm$ 4	31 $\pm$ 3	39 $\pm$ 4	44 $\pm$ 4
Normalized cell volume$^\mathrm{c}$	COND	98 $\pm$ 1	96 $\pm$ 1	94 $\pm$ 1	92 $\pm$ 1
(%)*	PAT	102 $\pm$ 2	111 $\pm$ 3	111 $\pm$ 3	110 $\pm$ 3

$^\mathrm{a}$Average of $n$ = 10 samples for femoral condyles and n=5 samples for patellae

$^\mathrm{b}$Average of $n$ = 946 signals from the 10 femoral condyles and $n$ = 751 signals from the 5 patellae

$^\mathrm{c}$Measured in a previous study (Madden et al. 2013)

## Discussion

The results of this study support previous observations that chondrocytes in the intact cartilage respond to mechanical compression by initiating intracellular calcium transients during and immediately following tissue compression (Han et al. 2012). A distinct trend was observed for an increase in calcium signaling activity with increasing compressive tissue strain ($p$ = 0.133, Fig. 5a), thus confirming our hypothesis. Previous findings suggested that, following an initial mechanical stimulus, cells may become desensitized to further signaling, therefore the results observed here may underestimate the relationship between tissue loading and calcium signaling (Donahue et al. 2003; Han et al. 2012). This desensitization may be explained in part by the lack or defficiency of certain signaling pathways in chondrocytes, such as the T-type voltage-gated calcium channels which are associated with repetitive signaling in other cells types (Lu et al. 2012). Stretch-activated ion channels (SACs) likely play a key role in the mechanobiological response of chondrocytes (Guilak et al. 1999; Roberts et al. 2001; Mobasheri et al. 2002), and it is possible that the majority of these channels are recruited during the initial mechanical loading step. Receptor desensitization and ion channel fatigue are also factors that may contribute to the diminishing response with repeated mechanical loading (Ralevic and Burnstock 1998).

The time-course response of chondrocyte calcium signaling observed here (76 $\pm$ 9 s after onset of loading ramp) agrees well with previous in situ work ($\sim$35 s; Han et al. 2012), and with observations of osmotically induced calcium signaling in intact mouse femora ($\sim$20 s; Clark et al. 2010) and calcium signaling following direct application of hydrostatic fluid pressure to isolated superficial zone bovine chondrocytes ($\sim$72 s; Mizuno 2005). Conversely, the peak calcium response of chondrocytes embedded in gel constructs occurs much more slowly ($\sim$200 s; Roberts et al. 2001). Therefore, while calcium signaling may be a downstream signaling event in cell-gel constructs, the results of the current study taken together with previous work on in situ chondrocytes (Clark et al. 2010; Han et al. 2012b) and isolated cells subjected to direct stimulation (Mizuno 2005) suggests that calcium signaling is an initial response in the chondrocyte mechanotransduction process in intact tissue. Since this response differs for cell-gel constructs, it is likely that interactions between the chondrocytes and their surrounding ECM plays a key role in this process. Furthermore, the majority of signaling observed here occurred during or immediately following the dynamic loading phase with minimal activity during the static hold period of each load (Fig. 4). Therefore, dynamic factors such as strain rate and fluid flow-induced shear stress may be more influential than loading magnitude in regulating the calcium response of chondrocytes to mechanical loading (Yellowley et al. 1997; Pingguan-Murphy et al. 2006). Primary cilia and transmembrane proteins such as integrins facilitate chondrocyte-ECM interaction, are highly responsive to fluid flow, and are directly involved in chondrocyte calcium signaling (Degala et al. 2011; Wann et al. 2012). Additionally, aggrecan expression following mechanical compression, which has been linked to calcium signaling (Fitzgerald et al. 2004), is highest in areas of high interstitial fluid flow (Buschmann et al. 1999). Future studies may utilize chemicals, such as integrin-specific Arg-Gly-Asp (RGD) peptides (Wright et al. 1997; Degala et al. 2011), to investigate the specific roles of these cellular components, determine the extent to which cell-ECM interactions regulate calcium signaling, and to elucidate which channels are involved in the associated signaling pathways for chondrocytes in intact cartilage.

At 10 % applied tissue strain, Ca^2+^ signaling activity appeared generally scattered and disorganized, whereas at loads $\ge$20 % tissue strain calcium signals occurred during or immediately after the transient loading phase with minimal activity during the static hold period (Fig. 4). This apparent synchronization of Ca^2+^ activity suggests that loads $\ge$20 % nominal tissue strain may represent a mechanical threshold for chondrocytes with respect to cartilage homeostasis. It is interesting to note the significant increase in the fraction of calcium signals at 40 % tissue strain (Fig. 5b), which corresponds to supra-physiological loading conditions (Herberhold et al. 1999). This finding could indicate that large compressive strains beyond physiological levels result in abnormal metabolic activity of the chondrocytes. Future studies could investigate this hypothesis by monitoring the regulation of genes associated with calcium signaling such as aggrecan, type II collagen, link protein, and matrix metalloproteinases (Fitzgerald et al. 2004) under the mechanical loads applied here to determine if the metabolic response is primarily anabolic or catabolic.

The population response of in situ chondrocytes under mechanical compression may also be related to the superficial zone ECM strain under mechanical compression. In a previous study under similar experimental conditions, the local ECM strain in the superficial zone of patellar cartilage was found to be greater than that in femoral condyles for a given nominal tissue strain (Table 1; Madden et al. , 2013). In the current study, patellar samples exhibited a trend toward a greater percentage of cells responding with calcium signals for a given tissue load compared to femoral condyles (Fig. 5a), although these differences were not statistically significant likely due to the relatively small number of patellar samples. This observation provides further evidence implicating transmembrane proteins and receptors that interact with the ECM in the calcium response of chondrocytes in intact cartilage, as suggested by others (Guilak et al. 1999; Millward-Sadler et al. 1999; Loeser 2002; Mobasheri et al. 2002; Millward-Sadler and Salter 2004; Degala et al. 2011, 2012; Wann et al. 2012).

Furthermore, calcium signaling has been linked to changes in cell volume and membrane expansion (Guilak et al. 2002; Chao et al. 2006). We have shown distinctly different chondrocyte deformations between femoral condyle and patellar samples (Table 1). More specifically, transverse (width, depth) strains are much higher in patellar cells compared to femoral condyle cells under equivalent compressive tissue loads (Madden et al. 2013). This could lead to differences in membrane expansion and unfolding events, both of which may be associated with calcium signaling via SACs that are activated primarily by membrane tension (Sachs 2010). Differences in cytoskeletal structure, which varies with depth and between load-bearing and non-load-bearing regions of joints (Eggli et al. 1988; Durrant et al. 1999), may also contribute to the regional differences in calcium signaling. Since integrin signaling is closely associated with the cell cytoskeleton, cytoskeletal differences could account for some of the differences in calcium signaling observed here (Loeser 2002; Millward-Sadler and Salter 2004). Patellar cartilage is also more permeable than femoral condyle cartilage (Froimson et al. 1997). This may result in differences in the fluid-velocity profile during the transient loading phase, which influences chondrocyte calcium signaling (Degala et al. 2012). The pericellular matrix (PCM) of cells affects the fluid flow around chondrocytes, thus differences in PCM structure and mechanical properties might also contribute to any differences between joint regions both in terms of deformation and calcium signaling (Guilak et al. 2006; Madden et al. 2013). Differences in chondrocyte cytoskeletal structure and PCM characteristics between joint regions should be investigated in future studies.

Calcium signal characteristics were moderately affected by the magnitude of compressive loading. Signal amplitude remained relatively consistent across all tissue loads for both joint regions, with the patellar cells exhibiting significantly shorter signaling events for the 10–30 % tissue strain conditions (Fig. 6). The lack of a significant difference at 40 % tissue strain, despite marked differences in local mechanics, may again be a reflection of diminished mechanosensitivity (Donahue et al. 2003; Han et al. 2012). Calcium signal amplitude is a reflection of changes in intracellular Ca^2+^ and can provide clues as to the function of the calcium signal. For example, IP$_3$ pathway opening is enhanced by modest increases in intracellular Ca^2+^ and inhibited by relatively large increases in Ca^2+^ (Berridge 1993; Bootman et al. 2001). The consistently higher signal amplitude in femoral condyle cells might indicate greater access to intracellular calcium stores. The decrease in signal amplitude at 40 % nominal tissue strain was observed for both joint regions and may reflect a depletion of these intracellular calcium stores.

The duration of calcium signals remained consistent across all applied loads for femoral condyle cells. For patellar chondrocytes, there was an increase in signal duration with increasing load (Fig. 6). It has been shown that calcium signal duration differentially regulates specific genes in neuronal-like CA77 cells (Durham and Russo 2000). However the role of signal duration in chondrocytes remains unclear. Duration is associated with the function of a calcium signal in terms of the distance to effector systems; longer signals can reach effector systems further away from the signal origin (Berridge et al. 2003). It is unlikely that the temporal differences observed here (5–10 s) would significantly alter the effector systems reached as some calcium signals can be on the order of 10^-6^ $\upmu$s in other cell types (Berridge et al. 2003). Instead, the temporal differences between femoral condyle and patellar chondrocyte calcium signaling events may reflect signal tuning as a result of differing functional requirements and loading patterns of the respective cartilages, where structural differences have likely evolved and influence local cartilage properties (Little and Ghosh 1997; Herzog et al. 1998; Jurvelin et al. 2000; Treppo et al. 2000). Indeed, aggrecan synthesis rates differ between joint regions (Little and Ghosh 1997) and are associated with intracellular calcium (Fitzgerald et al. 2004). At 40 % nominal tissue strain, the local ECM strain in patellae (44 %) is much greater than in femoral condyles (34 %, Table 1) (Madden et al. 2013). The significant difference in signal duration observed at 40 % tissue strain may be a reflection of this large disparity in the local mechanical environments of the chondrocytes.

Compressive mechanical loading of intact articular cartilage resulted in calcium signals in the chondrocytes. The percentage of cells responding with calcium signaling tended to increase with increasing load magnitude ($p$ = 0.133). Patellar samples appeared to exhibit more calcium signaling cells at each tissue load compared to femoral condyles, although this result was not significant likely due to the small number of patellae tested. The response times for calcium signaling agreed well with previous in situ studies, and they were faster than times reported for cell-gel constructs. This study provides new insight into calcium signaling in the intact cartilage attached to its native bone and further emphasizes the importance of studying chondrocytes in their natural environment. Future work using this in situ approach should investigate the anabolic or catabolic nature of the cellular response as it relates to calcium signaling and loading magnitude. Further studies will investigate the effect of loading magnitude on the calcium signaling behavior of chondrocytes from osteoarthritic cartilage, which undergo greater deformation than those in healthy tissue (Han et al. 2010). Currently, OA is a prevalent and irreversible joint disease with limited clinical treatment options. These results present an important step toward understanding the mechanisms underlying possible Ca^2+^-dependent signaling pathways that may be involved in cartilage homeostasis and the onset and progression of OA.
